# High Resolution Melting Analysis: A Rapid Screening and Typing Tool for Common β-Thalassemia Mutation in Chinese Population

**DOI:** 10.1371/journal.pone.0102243

**Published:** 2014-08-04

**Authors:** Min Lin, Ji-Wei Jiao, Xiu-Hui Zhan, Xiao-Fen Zhan, Mei-Chen Pan, Jun-Li Wang, Chun-Fang Wang, Tian-Yu Zhong, Qin Zhang, Xia Yu, Jiao-Ren Wu, Hui-Tian Yang, Fen Lin, Xin Tong, Hui Yang, Guang-Cai Zha, Qian Wang, Lei Zheng, Ying-Fang Wen, Li-Ye Yang

**Affiliations:** 1 Central Laboratory, Chaozhou Central Hospital Affiliated to Southern Medical University, Chaozhou, Guangdong Province, People's Republic of China; 2 Medical Laboratory, First Affiliated Hospital of Shantou University Medical College, Shantou, Guangdong Province, People's Republic of China; 3 Department of Biology, Hanshan Normal University, Chaozhou, Guangdong Province, People's Republic of China; 4 Department of Medical Laboratory, Hospital of Youjiang Medical University for Nationalities, Baise, Guangxi Province, People's Republic of China; 5 Medical Laboratory, First Affiliated Hospital of Gannan Medical University, Ganzhou, Jiangxi Province, People's Republic of China; 6 Laboratory Medical Center, People's Hospital of Yunan Province, Kunming, Yunnan Province, People's Republic of China; 7 Department of Medical Laboratory, Chengdu Women's & Children's Central Hospital, Chengdu, Sichuan Province, People's Republic of China; 8 Laboratory Medical Center, Nanfang Hospital, Southern Medical University, Guangzhou, Guangdong Province, People's Republic of China; 9 Medical Laboratory, Meixian People's Hospital, Meizhou, Guangdong Province, People's Republic of China; Chang Gung University, Taiwan

## Abstract

β-thalassemia is a common inherited disorder worldwide including southern China, and at least 45 distinct β-thalassemia mutations have been identified in China. High-resolution melting (HRM) assay was recently introduced as a rapid, inexpensive and effective method for genotyping. However, there was no systemic study on the diagnostic capability of HRM to identify β-thalassemia. Here, we used an improved HRM method to screen and type 12 common β-thalassemia mutations in Chinese, and the rapidity and reliability of this method was investigated. The whole PCR and HRM procedure could be completed in 40 min. The heterozygous mutations and 4 kinds of homozygous mutations could be readily differentiated from the melting curve except c.-78A>G heterozygote and c.-79A>G heterozygote. The diagnostic reliability of this HRM assay was evaluated on 756 pre-typed genomic DNA samples and 50 cases of blood spots on filter paper, which were collected from seven high prevalent provinces in southern China. If c.-78A>G heterozygote and c.-79A>G heterozygote were classified into the same group (c.-78&79 A>G heterozygote), the HRM method was in complete concordance with the reference method (reverse dot blot/DNA-sequencing). In a conclusion, the HRM method appears to be an accurate and sensitive method for the rapid screening and identification of β-thalassemia mutations. In the future, we suggest this technology to be used in neonatal blood spot screening program. It could enlarge the coverage of β-thalassemia screening program in China. At the same time, its value should be confirmed in prospectively clinical and epidemiological studies.

## Introduction

Beta-thalassemia (β-thalassemia) is a group of single-gene genetic disorders resulting from more than 200 point mutations in the hemoglobin-β gene (HBB) (MIM# 141900; GenBank genomic reference sequence NC_000011.9) on chromosome 11 [1]. It is prevalent in Mediterranean countries, the Middle East, Central Asia, India, Southern China, and the Far East as well as countries along the north coast of Africa and South America. The high gene frequency of β-thalassemia in these regions is most likely related to the selective pressure from Plasmodium falciparum [2], [3]. World Health Organization estimated that about 1.5% of the global population (80 to 90 million people) were carriers of β-thalassemia, with about 60,000 symptomatic individuals born annually, the great majority in the developing world [4]. Patients of each ethnic population carry their own specific types of mutations, including a few very common ones and a variable number of rare ones [1], [4]. To date, 45 single-nucleotide mutations and small deletions in HBB have been reported in Chinese, more than 90% mutation of those are c.-78A>G, c.52A>T, c.126_129delCTTT, c.216_217insA and c.316-197C>T [5].

Various methods have been developed to detect β-thalassemia mutations, these include allele-specific oligonucleotide (ASO) blot, reverse dot blot (RDB), microarrays, PCR-single strand conformational polymorphism analysis (PCR-SSCP), PCR-DNA sequencing and melting probe (MeltPro) assay [6], [7]. Among these methods, RDB analysis is the only method approved by China SFDA (China Food and Drug Agency) and used in the clinical diagnosis. We have developed a gene chip by RDB assay combined with a flow-through hybridization technology platform previously, which decreased the time of the hybridization process from 5 or 6 h to 1.5 h [8]. However, the RDB is still expensive, time-consuming and low throughput in large scale screening.

High-resolution melting (HRM) analysis is a new and rapid method for mutation screening in which PCR and mutation scanning are performed simultaneously in a single procedure within 40 minutes. Sensitivity and specificity of HRM for mutation detection are extremely high, and this technique also has the advantages of low cost and high throughput. Recently, this technique has also been used in the detection of α- and β-thalassemia [9], [10], [11], but there is no systemic study on the diagnostic capability of HRM to identify β-thalassemia. In the previous reports, the major interference was the SNP in hemoglobin-β gene such as rs713040, rs10768683 and rs1609812, which reduced the accuracy. Therefore, HRM method for routine β-thalassemia carrier screening must overcome those obstacle.

In this study, we developed an HRM assay to genotype the common Chinese β-thalassemia mutations rapidly and effectively. To assess the clinical value of this test, we conducted validation study including 756 pre-typed genomic DNA samples and 50 cases of blood spots on filter paper, which collected from seven high prevalent provinces in southern China.

## Materials and Methods

### Patient samples

#### (1) Pre-genotyped DNA

**Figure 1:**
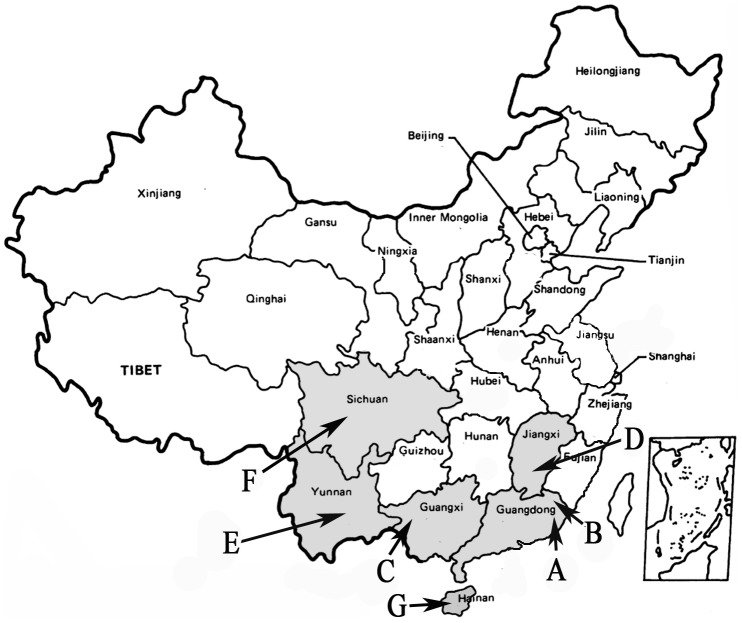
Geographic location of seven regions in China. A: Chaozhou area of Guangdong province; B: Meizhou area of Guangdong province; C: Baise area of Guangxi Zhuang Autonomous Region; D: Ganzhou area of Jiangxi province; E: Dai Autonomous Prefecture of Xishuangbanna of Yunan province; F: Chengdu area of Sichuan province; G: Li Autonomous Prefecture of Lingshui of Hainan province.

a total of 756 genomic DNA samples were used, which included 466 β-thalassemia minor, 18 β-thalassemia major, 17 cases of Hb J-Bangkok (HBB: c.170G>A), 15 cases of Hb NewYork (HBB: c.341T>A) and 240 normal samples. These samples were collected from six provinces in southern China as follows: Chaozhou Central Hospital (A, Guangdong province), Meixian People's Hospital (B, Guangdong province), Hospital of Youjiang Medical University for Nationalities (C, Guangxi province), Affiliated Hospital of Gannan Medical University (D, Jiangxi province), First People's Hospital of Yunan Province (E, DNA sample collected from Dai Autonomous Prefecture of Xishuangbanna of Yunan province), Sichuan Academy of Medical Science &Sichuan Provincial People's Hospital (F, Sichuan province) and Chaozhou Hybribio Limited Corporation (G, DNA sample collected from Li Autonomous Prefecture of Lingshui of Hainan Province), which have been identified by RDB and PCR-DNA sequencing in our previous study or local hospitals [8], [12]. The geographical location and detailed messages of these samples were shown in Fig.1 and Table 1, respectively. These 756 genomic DNA were extracted from 725 cases of blood, 5 cases of amniotic fluid, 12 cases of chorionic villus and 14 cases of cord blood using a DNA blood mini kit (QIAGEN China Shanghai Co., Ltd). The DNA concentration was determined by UV-2000 spectrophotometer (UNICO Shanghai Instruments Co., Ltd). All DNA templates were adjusted to about 20 ng/µL concentration. These samples were stored at −80°C until use.

**Table 1:** Information of 756 pre-typed DNAs and 50 blood spots in the verification test.

Mutation types	Pre-type DNA								Blood spots
	*n*	Han^A^ (*n*)	Han^B^ (*n*)	Zhuang^C^ (*n*)	Han^D^ (*n*)	Dai^E^ (*n*)	Han^F^ (*n*)	Li^G^ (*n*)	Han^A^ (*n*)
**β-thalassemia minor**									
c.-79A>G	12			7	4	1			3
c.-78A>G	56	9	7	12	17	10	1		5
c.45_46insG	6		2	4					
c.52A>T	63		1	39		21	2		4
c.79G>A	71	5	7	5	8	45	1		1
c.84_85insC	4			4					
c.92+1G>T	7			7					
c.126_129delCTTT	162	34	19	25	14	32	3	35	12
c.130G>T	9			5		3	1		
c.216_217insA	10			7	1	2			
c.316-197C>T	66	21	15	14	12		3	1	15
**β-thalassemia major**									
c.126_129delCTTT/c.126_129delCTTT	3			2				1	
c.316-197C>T/c.316-197C>T	2			2					
c.52A>T/c.52A>T	1			1					
c.84_85insC/c.84_85insC	1				1				
c.126_129delCTTT/c.316-197C>T	8	2	1	5					
c.126_129delCTTT/c.-78A>G	1			1					
c.52A>T/c.79G>A	1			1					
c.52A>T/c.216_217insA	1			1					
**Hb Variants**									
c.170G>A (Hb J-Bangkok)	17	3	7	2	5				
c.341T>A (Hb NewYork)	15	2	12	1					
**Health subjects**		240							10
**Total**	756	316	71	145	62	114	11	37	50

Han^A^: Han Chinese from Chaozhou area of Guangdong province; Han^B^: Han Chinese from Meizhou area of Guangdong province; Zhuang^C^: Zhuang ethnic group from Baise area of Guangxi Zhuang Autonomous Region; Han^D^: Han Chinese from Ganzhou area of Jiangxi province; Dai^E^: Dai ethnic group from Dai Autonomous Prefecture of Xishuangbanna of Yunan province; Han^F^: Han Chinese from Chengdu area of Sichuan province; Li^G^: Li ethnic group from Li Autonomous Prefecture of Lingshui of Hainan province.

#### (2) Blood spots

40 cases of β-thalassemia minor and 10 cases of normal control, which have been identified by RDB in Chaozhou Central Hospital, attended our study (Table 1). Blood spots were collected from finger-prick blood onto 3 MM S&S903 filter paper (Whatman) and were dried at ambient temperature. The dried blood spots were stored individually in zip-lock bags containing silica desiccant beads and refrigerated (4–8°C) whenever possible use.

These samples of unrelated thalassemia patients and normal controls were used to test the specificity and accuracy of this assay in a blind study. All studies were approved by the Ethics Committee of Chaozhou Central Hospital Affiliated to Southern Medical University, Ethics Committee of Hospital of Youjiang Medical University for Nationalities, Ethics Committee of First Affiliated Hospital of Gannan Medical University, Ethics Committee of People's Hospital of Yunan Province, Ethics Committee of Chengdu Women's & Children's Central Hospital and Ethics Committee of Meixian People's Hospital. Information sheets with nationality, sex, age, natives or not and written consent forms were available in Chinese to ensure comprehensive understanding of the study objectives, and informed consent was signed or thumb printed by the participants.

### DNA isolation from the blood spots

DNA was isolated from the whole blood impregnated filter paper with the rapid Chelex-100 method as described previously [13], [14]. Two pieces of 3-mm diameter circle were punched out from the dried blood spot into a 1.5-mL microcentrifuge tube. 500 µL of sterile millipore water was added to the tube, then the sample was vortexed at room temperature for 30 min. The samples were centrifuged for 3 min at 14,000 rpm, most of the supernatant was removed, and 160 µL of 10% Chelext-100 resin (Bio-Rad Laboratories, Hercules, CA) was added. The tubes were incubated in a MJ Mini Personal Thermal Cycler (Bio-RAD Company) for 120 min at 56°C followed by 10 min incubation at 99°C. After cooling, the samples were centrifuged for 2–3 min at 14,000 rpm. The supernatant was used as DNA templet. All DNA samples were adjusted to 20 ng/µL concentration by UV-2000 spectrophotometer and stored at −80°C.

### Construction of standard plasmids as the reference molecule

Standard mutation plasmids were constructed by site-specific mutagenesis technology in our previous study [8], which included 12 kinds of β-thalassemia mutations (c.-79A>G, c.-78A>G, c.45_46insG, c.52A>T, c.79G>A, c.84_85insC, c.126_129delCTTT, c.130G>T, c.216_217insA, c.92+1G>T, c.92+5G>C, c.316-197C>T). Direct sequencing was performed by Shanghai Invitrogen Biotechnology Co., Ltd. (Shanghai, China) to confirm the sequence of these mutations, which were shown in our previous report [8]. The extracted plasmids was diluted with the appropriate volume of a TE buffer solution to several concentration (10^9^–10^2^ copy/mL), and stored at −20°C until use. The concentration of pUC18 was measured by UV absorption at 260 nm. We used wild type DNA sample (identified in previous studies) and/or the mutation plasmids to prepare various kinds of genotype, including heterozygote and homozygote.

### Design of primers

We used Oligo 6.64 (Molecular Biology Insights) and Primer Premier 5.0 (Premier Biosoft) software for primer design. Six PCR primer pairs were designed to amplify the regions which encompass the sites of 12 kinds of known mutations occurring in hemoglobin-β gene in the Chinese population (NCBI Reference Sequence: NG_000007.3). For increasing their accuracy and convenience of clinical application, some principles were carried out as follows: (1) the annealing temperature (Tm) of all primers was designed in the same level; (2) six β-thalassemia mutation including c.-78A>G, c.52A>T, c.79G>A, c.126_129delCTTT, c.216_217insA and c.316-197C>T, which account for over 90% of β-thalassemia mutations in Chinese [5], were designed to locate in different amplicons; (3) In our previous investigation, we found that two SNP (rs713040, c.9 T>G and rs1609812, c.316–185 C>T) and a kind of Hb variant (Hb J-Bangkok) could produce interference for identification of β-thalassemia mutations by HRM analysis. The heterozygous frequencies of rs1609812 ranged from 46.3% to 52.9% in Chinese population (http://www.ncbi.nlm.nih.gov/projects/SNP/snp_ref.cgi?rs=1609812). The heterozygous frequency of rs1609812 was 55.8% in Chinese population (http://www.ncbi.nlm.nih.gov/projects/SNP/snp_ref.cgi?rs=713040). In order to solve this problem, we designed two primers to overlay these locations to block these interferences. The principle of this technology was shown in Appendix S1. All information of the primers was listed in Table 2. The primers synthesized were all of standard molecular biology quality (Shanghai Invitrogen Biotechnology Co., LTD).

**Table 2:** The primers for HRM analysis of 12 kinds of β-thalassemia mutations.

Name	Sequence 5′-3′	Location	Product	Typing
HB01-F	AGGAGCAGGGAGGGCAG	NG_000007.3: 70483–70499	85 bp	c.-78&79 A>G Ht; c.-78A>G Ho
HB01-R	CAGTTGTGTCAGAAGCAAATGTA	NG_000007.3: 70545–70567		
HB02-F	ATGGTGCATCTGACTCCTGA	NG_000007.3: 70597–70619	80 bp	c.45_46insG Ht; c.52A>T Ht; c.52A>T Ho;
HB02-R	TCACCACCAACTTCATCC	NG_000007.3: 70659–70678		
HB03-F	TGAACGTGGATGAAGTTGGTG	NG_000007.3: 70652–70672	89 bp	c.79G>A Ht; c.84_85insC Ht; c.92+1G>T Ht; c.316-197C>T Ht;
HB03-R	TGCCCAGTTTCTATTGGTCTC	NG_000007.3: 70720–70740		
HB04-F	TATTTTCCCACCCTTAGGCT	NG_000007.3: 70802–70821	103 bp	c.126_129delCTTT Ht; c.130G>T Ht c.126_129delCTTT Ho;
HB04-R	TAGGGTTGCCCATAACAGCAT	NG_000007.3: 70884–70904		
HB05-F	GAAGGCTCATGGCAAGAAAG	NG_000007.3: 70909–70928	90 bp	c.216_217insA Ht;
HB05-R	TCACTCAGTGTGGCAAAGGT	NG_000007.3: 70979–70998		
HB06-F	TGCCTCTTTGCACCATTCTA	NG_000007.3: 71644–71663	82 bp	c.316-197C>T Ht; c.316-197C>T Ho;
HB06-R	GAAATATTTATATGCAGAGATATTGCTA	NG_000007.3: 71698–71725		

Ht: heterozygote; Ho: homozygote; c.-78&79 A>G Ht: c.-78A>G heterozygote or c.-79A>G heterozygote.

### PCR and HRM Assay

PCR reactions were performed on the LightCycler 480 II instrument (Roche Diagnostics) with the software LightCycler 480 Gene Scanning Software Version 1.5 (Roche Diagnostics). The PCR reaction was carried out in a total volume of 20 µL, containing 2 µL of sample DNA or standard genotype plasmids (1×10^5^ copies/well), 4 µL of 5×PCR buffer, 0.2 µL HotStar Taq, (Takara Biotechnology Dalian CO., China), 1 µL of 10 µmol/L each primer, 1.6 µL of 2.5 mM dNTP and 1 µL LC Green plus (Idaho Technology). In order to obtain information on melting profiles, standard control (wild type DNA/plasmid or plasmids) were used as reference genotypes for the HRM analysis. All DNA samples and standard control were amplified simultaneously in two parallel reactions with each primer set. The cycling conditions were the same for all amplicons as follows: 95°C for 3 min, followed by 40 cycles of 98°C for 10 s, 68°C for 10 s. Then the fragment were melted by raising the temperature as the following condition: denaturalization at 95°C for 1 min, renaturation at 40°C for 1 min, and then melting that consisted of a continuous fluorescent reading from 60 to 90°C at 25 acquisitions per °C.

Melting curve profiles were generated by increasing the temperature from 65°C to 95°C at a rate of 0.05°C/s, and fluorescence was continuously acquired. HRM analysis was performed using the software LightCycler 480 SW 1.5 (Roche Diagnostics). The software parameters including pre-melt slider settings, post-melt slider settings and threshold were given in Figure S1,S2, S3, S4, S5 and S6. Normalized melting curves showed the fluorescence signal against the temperature, and derivative plots showed the melting temperature peaks. The plasmids with known mutations were regarded as standard reference. When the plots of samples were classified into the standard reference, they were identified as the same genotype of the standard. The melting curves for primer settings HB01, HB02, HB03, HB04, HB 05 and HB06 could be differentiated into 3, 4, 5, 4, 2 and 3 groups, respectively.

### Statistical Analysis

The *χ* ^2^ test was used to evaluate whether a significant difference existed between the results obtained using the HRM assay and RDB analysis or DNA sequencing. Statistical analysis was performed with SPSS 16.0 software; *P*<0.05 was considered statistically significance.

## Results

**Figure 2:**
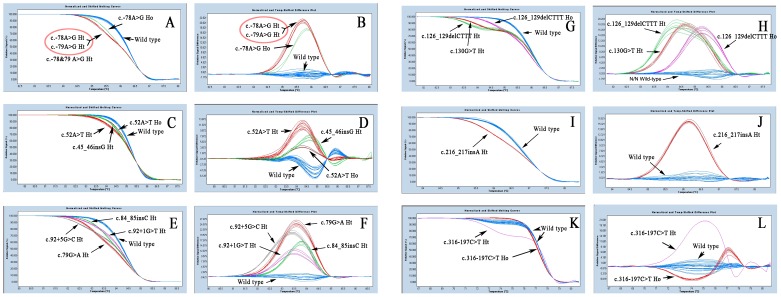
Screening and typing of 12 kinds of β-thalassemia mutations in Chinese by HRM analysis. The arrowheads indicated different genotypes. **A, C, E, G, I** and **K** were the Temp-shift melting curves; **B, D, F, H, J** and **L** were the Temp-shift difference plots. Ht: heterozygote; Ho: homozygote; c.-78&79 A>G Ht: c.-78 A>G heterozygote or c.-79A>G heterozygote. **A** and **B**: HB01 primer set for c.-78&79 A>G Ht and c.-78 A>G Ho; **C** and **D**: HB02 primer set for c.45_46insG Ht, c.52A>T Ht and c.52A>T Ho; **E** and **F**: HB03 primer set for c.79G>A Ht, c.84_85insC Ht, c.92+1G>T Ht and c.316–197C>T Ht; **G** and **H**: HB04 primer set for c.126_129delCTTT Ht; c.130G>T Ht and c.126_129delCTTT Ho; **I** and **J**: HB05 primer set for c.216_217insA Ht; **K** and **L**: HB06 primer set for c.316–197C>T Ht and c.316–197C>T Ho.

It took us about 40 minutes to complete PCR reaction and to differentiate common Chinese β-thalassemia mutations in a closed tube system. The melting curves of 12 kinds of β-thalassemia heterozygous mutations and 4 kinds of homozygous mutations (c.-78A>G, c.52A>T, c.126_129delCTTT, c.316–197C>T) were shown in Figure 2. All these heterozygous mutations and homozygous mutations could be easily distinguished from the normal control (wild type). On the other hand, these mutations could be identified from the melting curve except c.-78A>G heterozygote and c.-79A>G heterozygote (Figure 2A and Figure 2B).

Not agreed with Shih' report [11], c.-78A>G heterozygote and c.-79A>G heterozygote could not be distinguished by shape of melting curves in our study (Figure 2A and Figure 2B). The c.-78A>G homozygous mutation could be distinguished from c.-78A>G heterozygote and wild-type DNA (Figure 2A and Figure 2B). At the same time, we used the same primer set and the same PCR-HRM conditions described in Shih et al. 's study [11], c.-78A>G heterozygote and c.-79 A>G heterozygote could not be differentiated either. Because c.-79A>G heterozygote and c.-79A>G heterozygote were β^+^ thalassemia with similar clinical feature, we classified them into a group (c.-78&79 A>G) in the following validation test.

### Evaluation of interference factors

DNA sequencing results of 240 healthy subjects for hemoglobin-β gene indicated that the heterozygotic frequency of two common Chinese SNP [rs713040 (c.9T>G) and rs1609812 (c.316–185C>T)] were 44.58% (107/240) and 47.42% (109/240), respectively. Two primers sets (HB01 and HB06) were designed to block the two SNP interferences (Table 2), in the further study, melting curves of these 240 healthy subjects were identical, and didn't show any difference among them, indicated that the two interference SNPs were successfully blocked by our specific primers.

Hb J-Bangkok (c.170G>A) and Hb New York (c.341T>A) were the common Chinese Hemoglobin variants [12]. Primers set HB04 were designed to block the mutation of Hb J-Bangkok. Totally 17 cases of Hb J-Bangkok and 15 cases of Hb New York were used to evaluate the interference factor in our study. We could not find any difference between these samples and wild-type DNA from their melting curves. The results indicated that we successfully used primers set HB04 to block interferences of these two common Chinese hemoglobin variants.

### Validation of the assay

To validate our optimized experimental procedures, 756 pre-genotyped DNA and 50 dry blood spots were detected by this method in a blind assay (Table 1). Because c.316-197C>T was very rare in Chinese, we could not find this sample to do the verification test. Reference molecules (wild type, 12 kinds of β-thalassemia heterozygous mutations and 4 kinds of homozygous mutations) were also added in the same run. Comparing the normalized melting curve and the temp-shifted difference plot of each mutation generated from the reference samples, we were able to distinguish the unknown sample, mutant or wild type. If c.-78A>G heterozygote and c.-79A>G heterozygote were classified into a group (c.-78&79 A>G heterozygote), the concordance of HRM and RDB/DNA-sequencing was 100%. The sensitivity and specificity for these mutations detection were 100%.

No any difference was observed in the melting profiles of DNA extracted using the QIAGEN kit and the DNA extracted from the blood spots with Chelex-100.

The analytical sensitivity of the PCR-HRM assay was determined with plasmids carrying these 12 kinds of mutations. Our method was able to detect and distinguish as few as 1×10^2^ copies/mL of each plasmid mixed with equal normal genomic DNA. To evaluate the reproducibility of the PCR-HRM assay, 25 samples with different genotypes of thalassemia were detected for 5 times. These samples were successfully identified with their genotypes by HRM. No difference was observed among the 5 melting curves of each sample (100% specificity).

## Discussion

β-thalassemia is a common genetic disease in southern China, which produce major public health problem and social burden to the people in epidemic areas [12], [14], [15]. Although China health department and government had carried out many programs for prevention and control of thalassemia in some provinces (Guangdong and Guangxi), these programs could only cover a few prevalent area of southern China [16], [17]. Neonatal blood spot screening program for phenylketonuria (PKU), glucose-6-phosphate dehydrogenase deficiency (G6PD) and congenital hypothyroidism (CHT), which started in 1981 by China government, had been perfectly carried out in main rural areas and cities of China mainland [18], [19]. We try to combine β-thalassemia screening with neonatal blood spot screening program to enlarge the coverage of thalassemia screening program. Therefore, a rapid, cost-effective, high-throughput method to detect ten of thousands of dry blood spots was imperative. Traditional method of β-thalassemia screening including hematological parameters and hemoglobin electrophoresis could not be applied for dry blood spots of neonates. Some techniques could be used for screening β-thalassemia by dry blood spots, such as high performance liquid chromatography (HPLC) and capillary electrophoresis (CE) [20], [21], and amplification refractory mutation system-PCR, PCR-SSCP, denaturing high performance liquid chromatography (DHPLC), matrix-assisted laser desorption/ionization time of flight mass spectrometry (MALDI-TOF) and PCR-RDB [6]. However, these methods are time consuming and costly for large scale screening.

HRM analysis is a fast, cost-effective, and more convenient closed-tube genotyping approach for screening of genetic disorders [22], [23]. It not only could reduce the contamination risk, but also could be applied for high-throughput mutation scanning on genes for which large cohorts of patients has to be investigated. As early as 2008, Pornprasert S et al diagnosed some deletion forms and a few mutations of thalassemia by HRM analysis in Thailand successfully [9], [24], [25]. Recently, it has been used in the detection of β-thalassemia mutations in Chinese, but interference of SNP and Hb variants was still an embarrassment [10], [11].

In the study, we developed an optimized protocol for scanning the 12 common mutations causing β-thalassemia in Chinese. Different kinds of genotype combinations, which were prepared with standard plasmids and wild type DNA, could be correctly identified by melting curves. 756 samples were used for verification test. The samples were collected from four ethnic groups: Zhuang, Li, Dai and Han, which represented the major feature of β-thalassemia mutation in Chinese. Verification test showed the concordance of HRM and RDB/DNA-sequencing was 100%.

## Conclusions

The PCR-HRM method appears to be an accurate and sensitive method for the rapid screening and identification of β-thalassemia genotypes. In the future, we suggest using this technology in neonatal blood spot screening program. It could enlarge the coverage of β-thalassemia screening program in China. At the same time, its value should be confirmed in clinical and epidemiological studies prospectively.

## Supporting Information

Figure S1 **The software parameters of primer set HB01, including the pre-melt slider settings, post-melt slider settings and threshold.**
(JPG)

Figure S2 **The software parameters of primer set HB02, including the pre-melt slider settings, post-melt slider settings and threshold.**
(JPG)

Figure S3 **The software parameters of primer set HB03, including the pre-melt slider settings, post-melt slider settings and threshold.**
(JPG)

Figure S4 **The software parameters of primer set HB04, including the pre-melt slider settings, post-melt slider settings and threshold.**
(JPG)

Figure S5 **The software parameters of primer set HB05, including the pre-melt slider settings, post-melt slider settings and threshold.**
(JPG)

Figure S6 **The software parameters of primer set HB06, including the pre-melt slider settings, post-melt slider settings and threshold.**
(JPG)

Appendix S1 **The principle of the primers to block of SNP interferences.**
(DOC)
