# Supplementary figures and images for: High Resolution Melting Analysis: A Rapid Screening and Typing Tool for Common β-Thalassemia Mutation in Chinese Population
Source: PLoS One. 2014 Aug 4;9(8):e102243. doi: 10.1371/journal.pone.0102243 (PMC4121066; doi:10.1371/journal.pone.0102243)

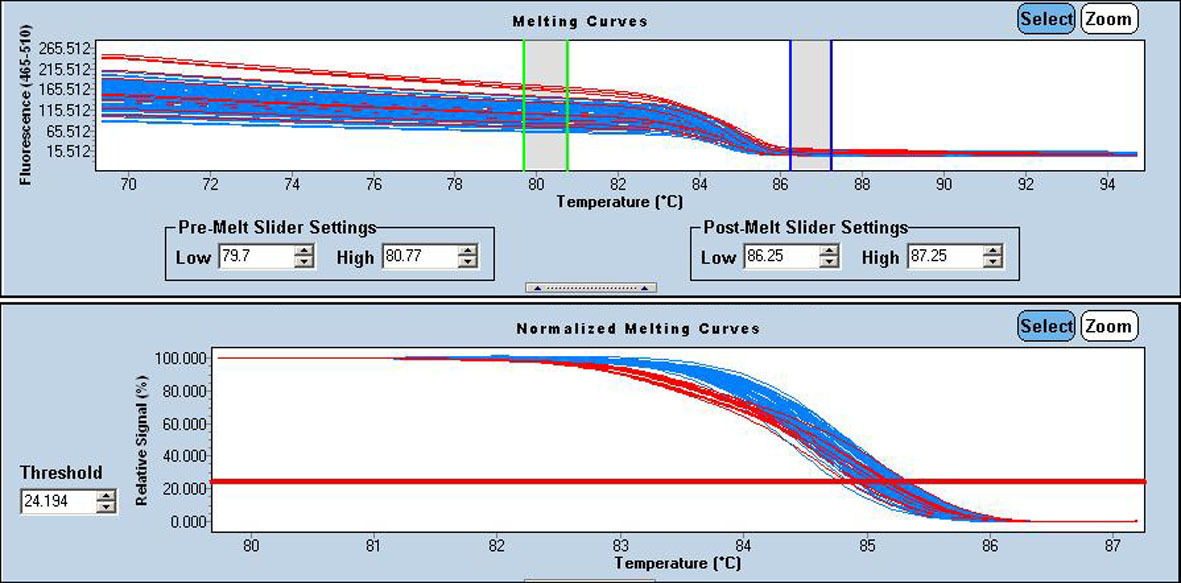

Supplement: Figure S1 — The software parameters of primer set HB01, including the pre-melt slider settings, post-melt slider settings and threshold. (JPG) [file pone.0102243.s001.jpg]

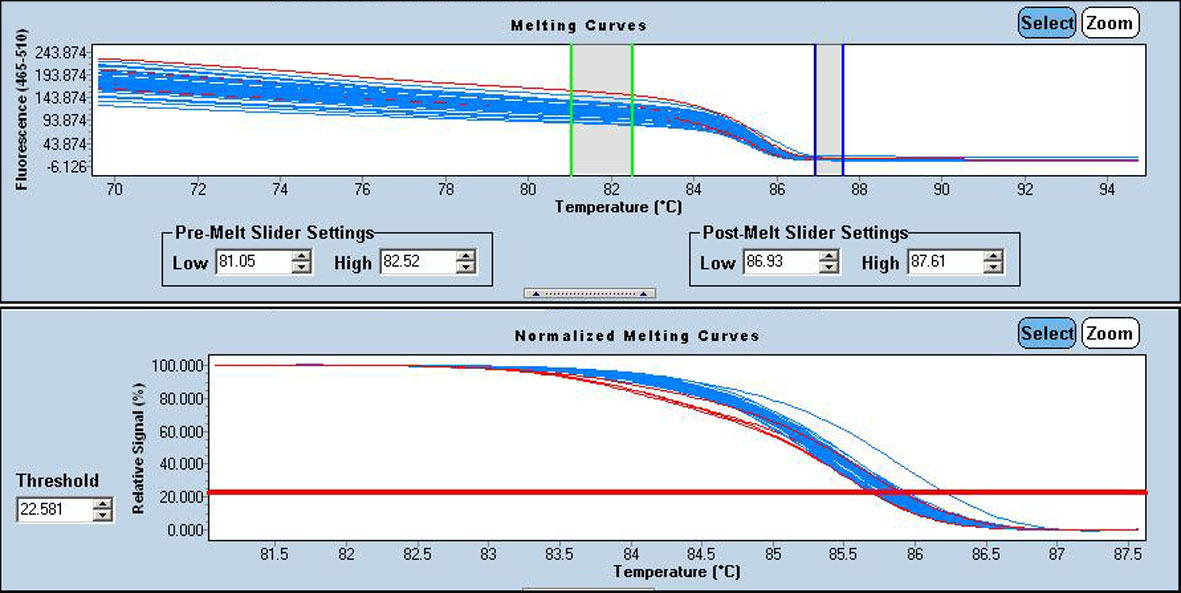

Supplement: Figure S2 — The software parameters of primer set HB02, including the pre-melt slider settings, post-melt slider settings and threshold. (JPG) [file pone.0102243.s002.jpg]

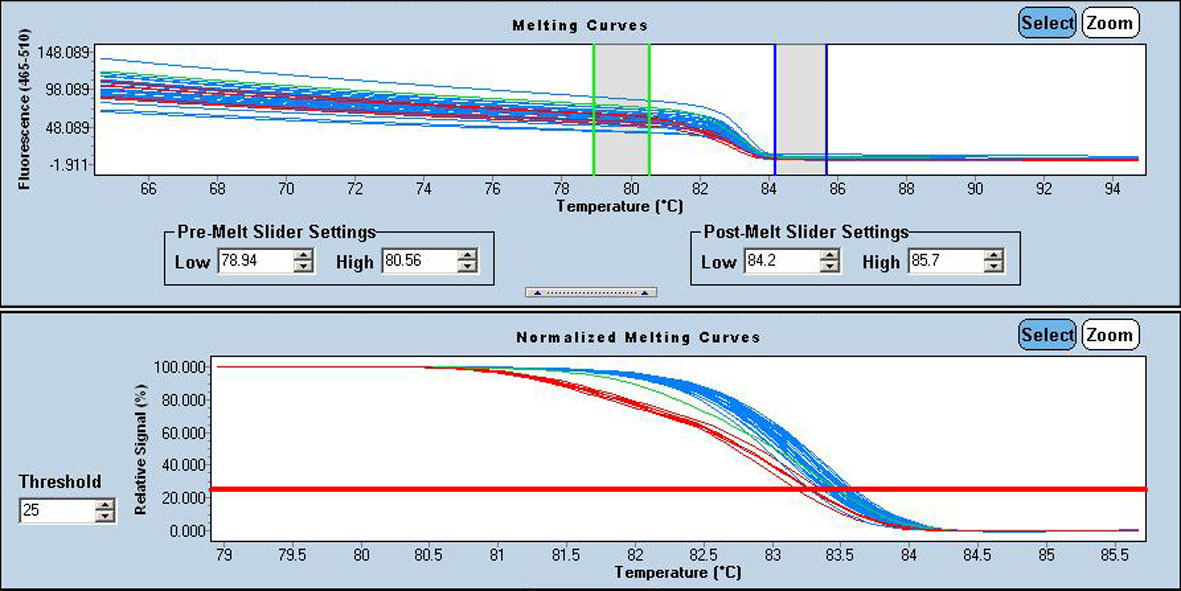

Supplement: Figure S3 — The software parameters of primer set HB03, including the pre-melt slider settings, post-melt slider settings and threshold. (JPG) [file pone.0102243.s003.jpg]

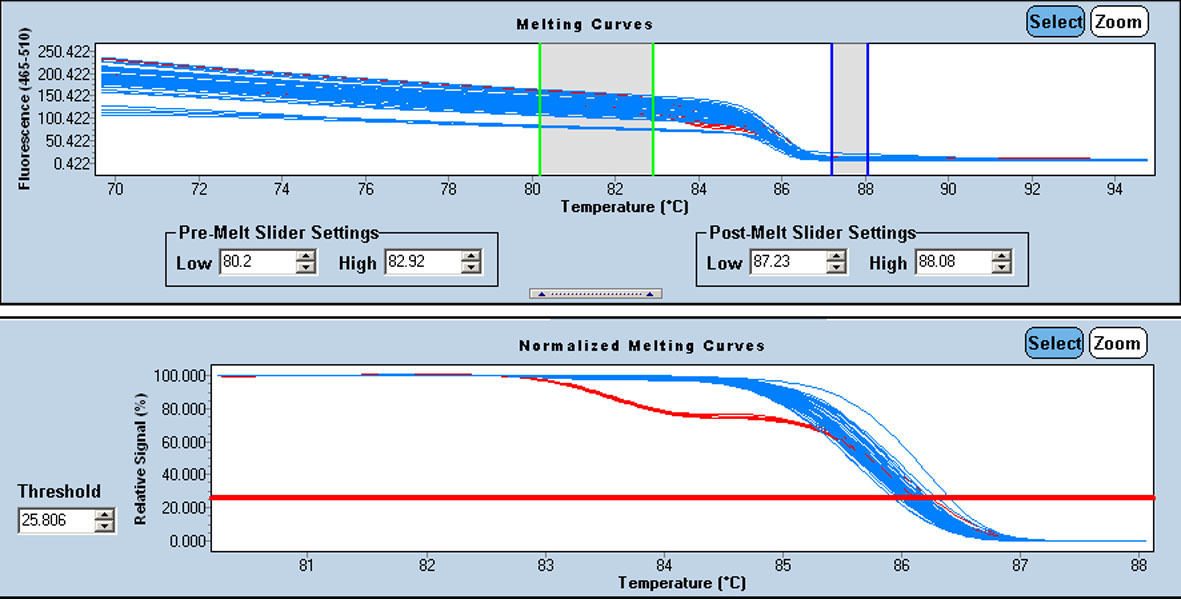

Supplement: Figure S4 — The software parameters of primer set HB04, including the pre-melt slider settings, post-melt slider settings and threshold. (JPG) [file pone.0102243.s004.jpg]

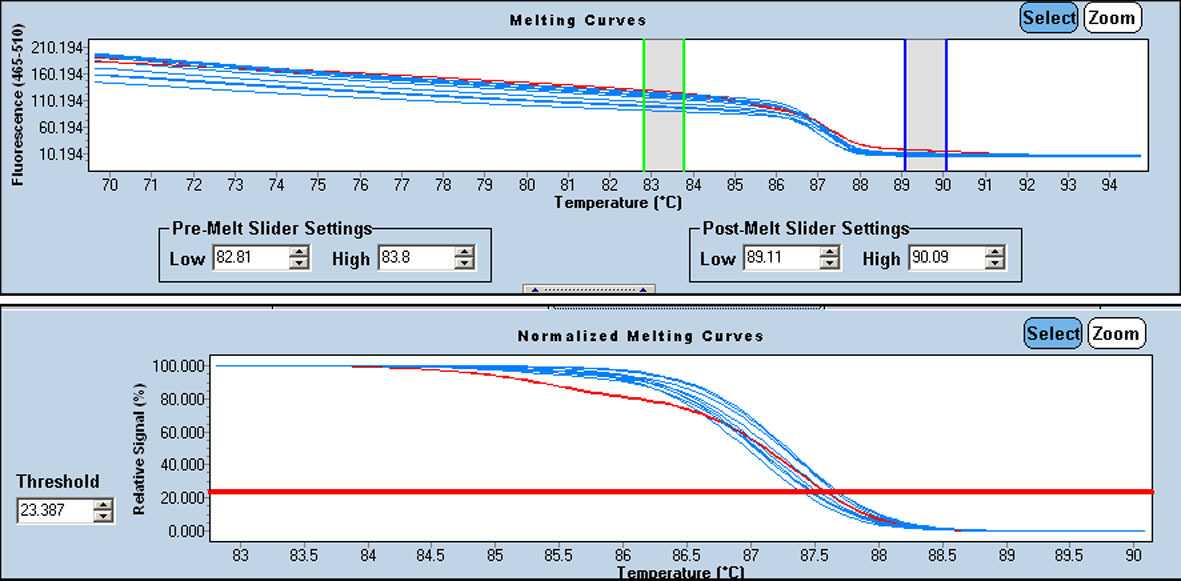

Supplement: Figure S5 — The software parameters of primer set HB05, including the pre-melt slider settings, post-melt slider settings and threshold. (JPG) [file pone.0102243.s005.jpg]

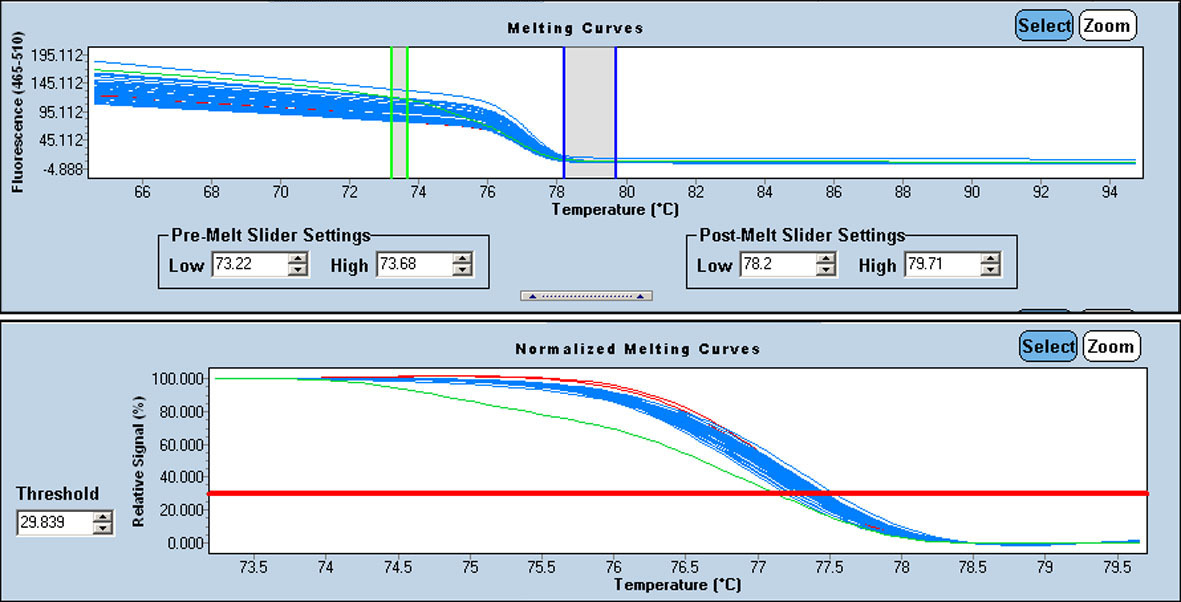

Supplement: Figure S6 — The software parameters of primer set HB06, including the pre-melt slider settings, post-melt slider settings and threshold. (JPG) [file pone.0102243.s006.jpg]
