# Supplementary material for: High Resolution Melting Analysis: A Rapid Screening and Typing Tool for Common β-Thalassemia Mutation in Chinese Population
Source: PLoS One. 2014 Aug 4;9(8):e102243. doi: 10.1371/journal.pone.0102243 (PMC4121066; doi:10.1371/journal.pone.0102243)
Supplement: Appendix S1 — The principle of the primers to block of SNP interferences. (DOC) [file pone.0102243.s007.doc]

This PCR technology has been used in some previous report, such as the article of Chang JG ^[1]^. The SNP rs713040 (c.9 T>C) was used as example to explain this technology. As shown in the Figure A, we designed the primer HB02-F to overlay the locations of SNP (rs713040, c.9 T>C). The primer HB02-F could combine with the wild-type DNA template completely. Although one base of primer HB02-F could not combine with mutation DNA temple, it didn’t affect PCR amplification. After one cycle, The PCR product was shown in Figure B. We could find a mismatch of PCR product of mutation DNA template, which located in the region of primer HB02-F. Then, the PCR product of mutation DNA template was used as PCR template in following PCR amplification (Figure C), but it could not be amplified. After 30 cycle of PCR amplification, the ratio of two kinds of PCR products (wild: mutation) was (2^30^-1): 1 (Figure C). Therefore, the influence of mismatch PCR product could be ignored in the further HRM analysis.

[1] Er TK, Kan TM, Su YF, Liu TC, Chang JG, et al (2012) High-resolution melting (HRM) analysis as a feasible method for detecting spinal muscular atrophy via dried blood spots. Clin Chim Acta 413:1781-1785.


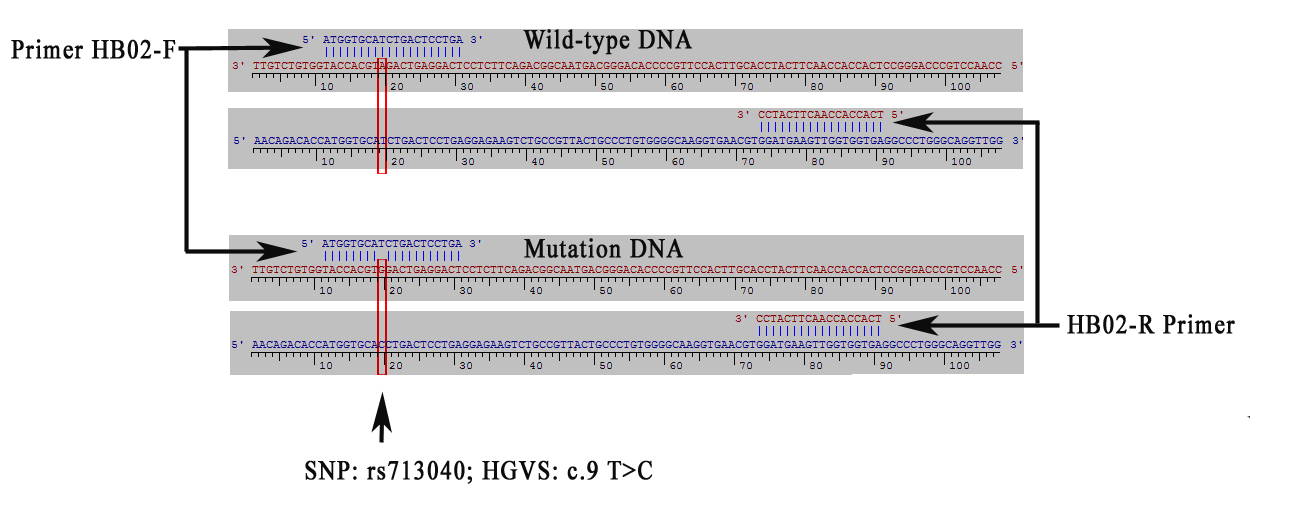


**Figure A**: Location of the primer setting HB02 in DNA template.


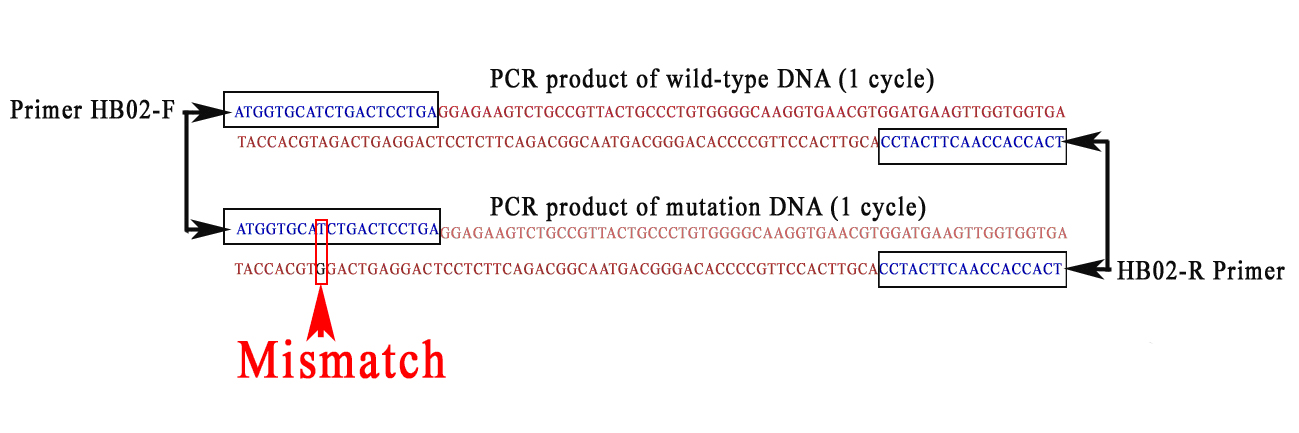


**Figure B**: The PCR product of primer setting HB02.


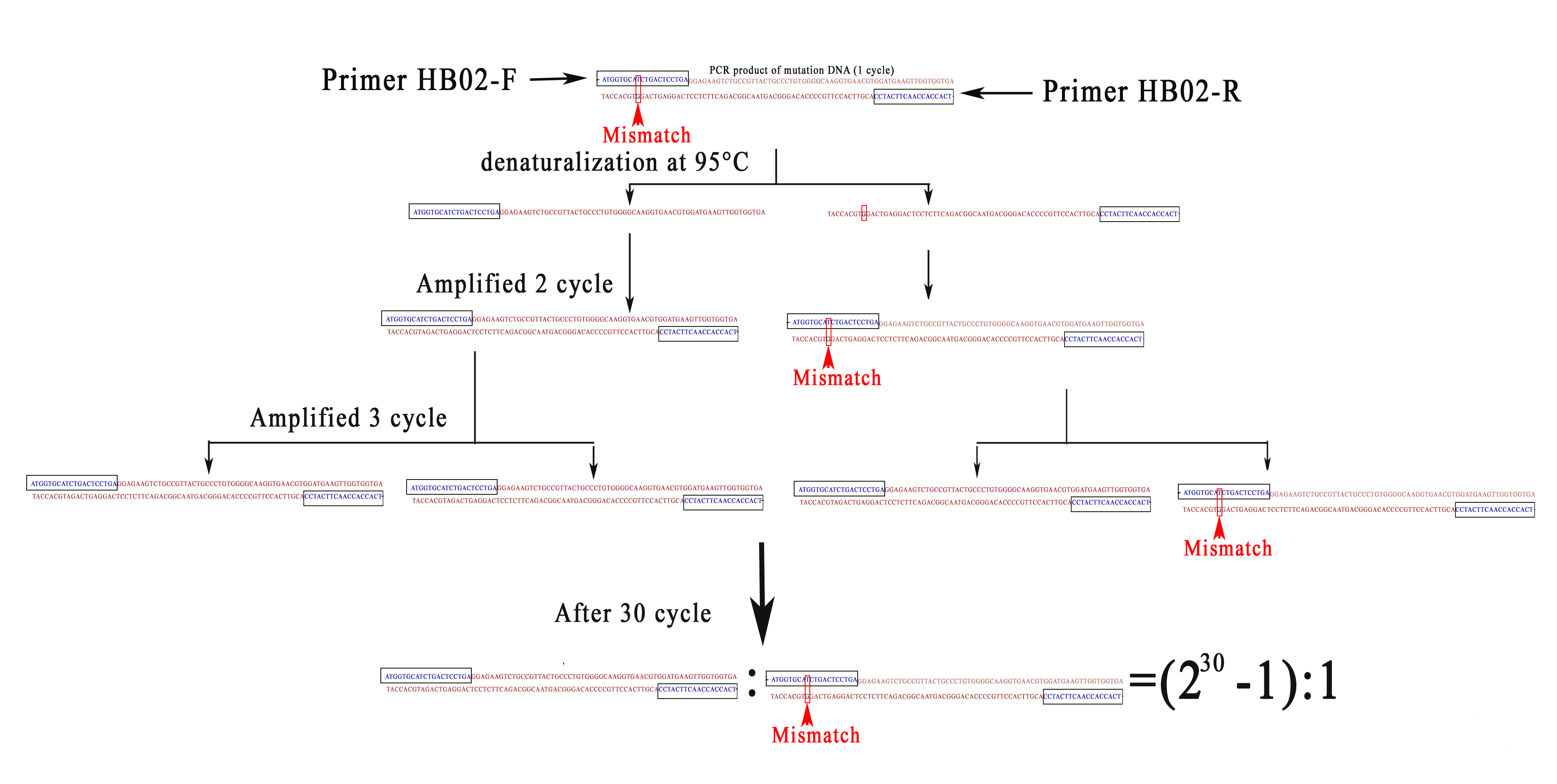


**Figure C**: The flow chat of PCR amplification.
